# Global prevalence of frailty in hemodialysis patients: a systematic review and meta-analysis

**DOI:** 10.3389/fmed.2025.1722657

**Published:** 2025-12-16

**Authors:** Ying Xiang, Jia-Le Tong, Weiwei Qian, Peng Deng

**Affiliations:** 1 Emergency Department of West China Hospital Sichuan University/West China School of Nursing, Sichuan University, Chengdu, China; 2 Disaster Medical Center, Sichuan University, Chengdu, China; 3 Nursing Key Laboratory of Sichuan Province, Chengdu, China; 4 Department of Emergency Medicine, West China Hospital, and Disaster Medical Center, Sichuan University, Chengdu, China

**Keywords:** frailty, hemodialysis, meta-analysis, prevalence, systematic review

## Abstract

**Background:**

The coexistence of frailty and hemodialysis is associated with a higher risk of adverse health outcomes, including hospitalization, mortality, and falls. Although the estimated prevalence of frailty in hemodialysis patients is widely reported, the results vary significantly across the relevant literature. Currently, comprehensive evidence regarding the global prevalence of frailty among hemodialysis patients remains unknown.

**Objective:**

In this systematic review and meta-analysis, our primary objective was to determine the global prevalence of frailty among hemodialysis patients, while accounting for stratification by sample size, age, duration of hemodialysis, gender, publication year, diagnostic criteria, and region.

**Design:**

A systematic review and meta-analysis.

**Data source:**

Cochrane Library, Medline, PubMed, Embase, Web of Science, Scopus, CINAHL, China Knowledge Resource Integrated Database (CNKI), Wanfang Database, Chinese Biomedical Database (CBM), and Weipu Database (VIP) from inception to 15 June 2023.

**Methods:**

Original articles that evaluated the prevalence of frailty in hemodialysis patients were included. Data extraction and methodological quality assessment of the included studies were performed independently by two reviewers. The pooled prevalence of frailty was estimated using the random-effects model. Meta-regression analysis and subgroup analysis were undertaken to explore sources of heterogeneity.

**Results:**

A total of 64 studies from 10 different countries met the inclusion criteria, with a total sample of 23,799 hemodialysis patients. The global pooled prevalence of frailty in hemodialysis patients was 39.6% (95%CI 35–44%), with significant heterogeneity across the various studies. The subgroup analysis results demonstrated that the prevalence of frailty among hemodialysis patients varied significantly based on sample size, age, duration of hemodialysis, gender, publication year, diagnostic criteria, and geographic region. Meta-regression showed that factors such as sample size, age, and gender independently emerged as predictors of frailty prevalence.

**Conclusion:**

High frailty prevalence was found in patients on hemodialysis, which may lead to poor health outcomes. The current analysis suggests that risk factors for frailty in hemodialysis patients should be further investigated in future studies, and early screening and interventions for frailty should be incorporated into the routine care of hemodialysis patients to reduce the negative impact of frailty.

**Systematic Review Registration:**

https://www.crd.york.ac.uk/PROSPERO/view/CRD42023411983, identifier PROSPERO (CRD42023411983).

## 1 Introduction

End-stage renal disease (ESRD) is a clinical syndrome involving an irreversible decline in kidney function, marked by the kidney’s inability to efficiently filter waste and maintain electrolyte balance, which has emerged as a significant global public health burden [(1); Cockwell et al., 2020]. It has been reported that ESRD leads to the annual mortality of approximately 1.2 million individuals, positioning it as the 8th leading cause of death (2, 3). Hemodialysis stands as the primary renal replacement therapy for patients diagnosed with ESRD, effectively contributing to the extension of patients’ life expectancy (4). According to the 2017 Global Burden of Disease, Injury, and Risk Factors Study, the global population of individuals living with hemodialysis was estimated to be around 3 million, with projections indicating a significant increase to 5.4 million by the year 2030 (5). Despite the alleviation of clinical symptoms and improved survival seen in ESRD patients undergoing hemodialysis, it is important to acknowledge that these patients still exhibit notably higher mortality and hospitalization rates compared to the general population (6, 7).

Frailty is a clinical syndrome caused by multiple factors, characterized by a decline in physiological reserves and reduced resistance to stress (8, 9). Frailty is prevalent in hemodialysis patients due to the adverse effects of metabolic disorders, toxin accumulation, and impaired physiological function induced by hemodialysis treatment (10, 11). Numerous studies have demonstrated that hemodialysis patients who suffer from frailty are more vulnerable to adverse health outcomes, such as falls, hospitalization, and death, thereby exacerbating the burden on both the families and society (12–14). Moreover, as the number of elderly hemodialysis patients increases dramatically with the aging of the population, the determination of frailty in this group is emerging as a novel concept and research hotspot with the potential to guide patients toward personalized treatment, thereby maximizing patient prognosis and quality of survival (15, 16). Last but not least, frailty is potentially reversible, which makes it the cornerstone for delaying the progression of frailty (17). Therefore, comprehensive estimates of the prevalence of frailty should be considered a high priority to reduce the risk of adverse events in hemodialysis patients.

Comprehending the current epidemiology of frailty in hemodialysis patients is crucial for clinical researchers to devise appropriate prevention and treatment strategies (18). Nevertheless, the existing literature regarding the prevalence of frailty among hemodialysis patients exhibits substantial variation owing to discrepancies in diagnostic criteria and sampling methods for frailty (6, 16). Two published systematic reviews have reported on the prevalence of frailty among hemodialysis patients (19, 20). After careful consideration, we have identified some common limitations as follows: (1) Only English language literature was included, limiting the extrapolation of the study; (2) Failure to rationally divide subgroups to explore differences in the prevalence of frailty among hemodialysis patients with different characteristics; (3) Sensitivity analyses were not conducted to assess the robustness of their findings. Therefore, we aimed to determine the global prevalence of frailty among hemodialysis patients as well as to explore the differences in the prevalence of frailty with different characteristics, in order to provide better guidance for healthcare professionals.

## 2 Methods

This systematic review and meta-analysis adhered to the guidelines outlined in the Preferred Reporting Items for Systematic Reviews and Meta-Analyses (21). The research protocol has been duly registered in the International Prospective Register of systematic reviews (CRD42023411983).

### 2.1 Search strategy and eligibility criteria

As the initial step, an exhaustive search was conducted in multiple databases, including the Cochrane Library, Medline, PubMed, Embase, Web of Science, Scopus, CINAHL, CNKI, WanFang, CBM, and VIP from their dates of inception through 15 June 2023. The initial keywords employed were “dialysis,” “hemodialysis,” “frail,” “frailty,” “frailties,” and “frailty syndrome.” In each database, keywords and medical subject headings (MeSH) were combined by using Boolean operators such as ‘and’ and ‘or’. The search strategy was meticulously drafted by the research team in collaboration with a specialist in medical statistics. Additionally, the reference lists of review papers were scrutinized to identify any additional pertinent studies. The detailed search strategy for each of the databases can be found in Supplementary Table S1.

To be eligible for inclusion, articles had to meet the following criteria: (1) they pertained to longitudinal or cross-sectional observational studies; (2) the participants, either overall or as a subsample, were hemodialysis patients; (3) frailty was defined using any authoritative diagnostic criteria; (4) sufficient data were reported to make it feasible to estimate the prevalence of frailty. The excluded criteria were: (1) reviews, comments, editorials, or conference abstracts; (2) articles lacking pertinent data; (3) duplicate publication of the relevant data; (4) studies with a sample size of less than 100; (5) literature in languages other than Chinese and English.

### 2.2 Data extraction and quality assessment

All search outcomes were input into reference management software for further analysis and organization. Data extraction was carried out by two reviewers who did a cross-check for accuracy after the extraction was complete. In cases where discrepancies arose between the two reviewers, a third reviewer was consulted to achieve consensus. The main variables extracted were: study characteristics (first author, publication year, country, region, study design, sample size), participant characteristics (age, duration of hemodialysis), the prevalence of frailty, and the diagnostic criteria for frailty. A few authors were contacted to supplement any missing information where possible.

Two reviewers carried out the methodological quality assessment independently, and any disagreements between them were clarified by discussion. Hoy’s risk of bias tool was applied to appraise the bias risk in each study (22). This tool comprises 10 items and assesses the quality of the literature in terms of both internal and external validity. Each item was marked with a score of 1 (yes) or 0 (no), and the overall rating is based on the cut-off values of 0–4, 5–7, and 8–10, representing high, moderate, and low risk of bias, respectively.

### 2.3 Data analysis

Data analyses were conducted by Stata 14.2 software, and a *p* value <0.05 (two-sided test) was considered statistically significance. Heterogeneity among included studies was assessed using the I2 statistic, which was classified into three categories based on *I*^2^ values: 25–50% (low), 50–75% (moderate), and ≥75% (high) (23). A random-effects model was employed to calculate the pooled prevalence of frailty if significant heterogeneity was detected; otherwise, a fixed-effects model was adopted. To test whether the results of the meta-analysis were robust, a sensitivity analysis was performed.

Subgroup analysis and meta-regression were undertaken to identify potential moderators of heterogeneity. The available literature data were categorized into subgroups based on the following criteria: sample size (≤300, >300), age (<60 years, ≥60 years), duration of hemodialysis (≤1 year, >1 year), gender (male, female), publication years (<2017, 2017 ~ 2020, 2021 ~ 2023), frailty criteria (FP, FRAIL, CFS, EFS, TFI, other criteria), regions (America, Asia, Europe). In each subgroup, we then computed the pooled estimates of frailty prevalence along with 95% confidence intervals. Publication bias was assessed by using a visualized funnel plot and objectively using Egger’s linear regression method. Additionally, the trim and fill analysis was also performed to adjust for any publication bias.

## 3 Results

### 3.1 Search outcome

**Figure 1:**
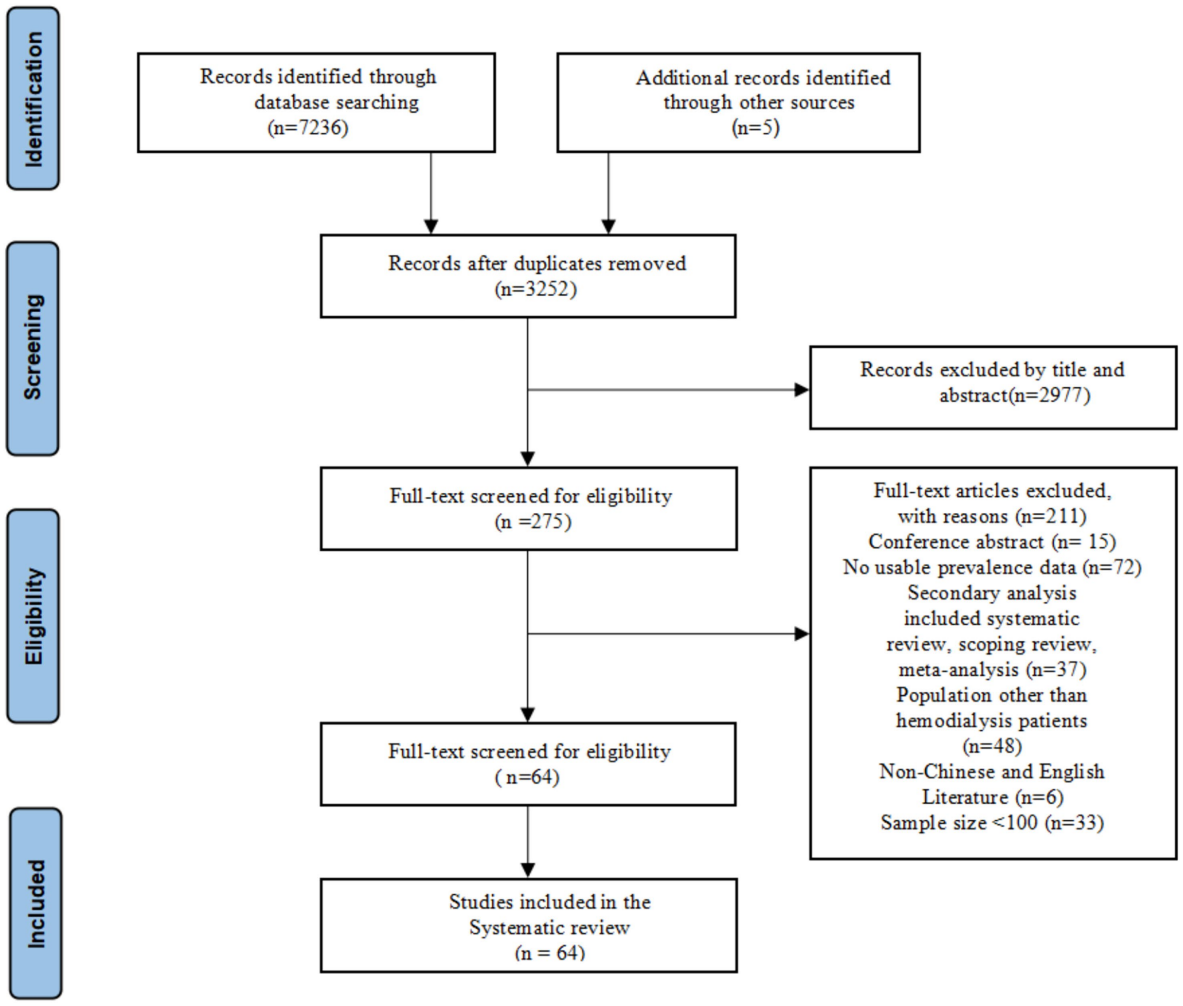
Flow diagram of study selection.

The initial electronic database search retrieved 7,236 articles, and an additional 5 studies were yielded from reviewing reference lists. After removing 3,989 duplicates, a total of 3,252 articles underwent screening based on their titles and abstracts, resulting in 275 studies that met the validation criteria. Of the 275 articles, a total of 64 studies were ultimately included in this study after the full-text examination. The flow chart depicting the selection process is detailed in Figure 1.

### 3.2 Characteristics of the included studies

The characteristics of the 64 studies that were eligible for inclusion are shown in Table 1. All studies were published in either English or Chinese and spanned from 2013 to 2023. The included studies were conducted in 10 different countries, and most of them came from Asia (*n* = 44, 68.8%), the rest were from America (*n* = 12, 18.8%) and Europe (*n* = 8, 12.5%). A total of 23,799 participants were recruited in the 64 included studies, with a range from 100 to 2,404. As reported in 64 studies, 38 were cross-sectional, and 26 were based on baseline data from cohort studies. The prevalence of frailty in hemodialysis patients was reported in all studies that met the inclusion criteria, ranging from 5.9 to 82.0%. The main diagnostic criteria that were accustomed to identify frailty included the Clinical frailty scale (CFS), Frailty Phenotype (FP), Frailty Index (FI), Fatigue, Resistance, Ambulation, Illness, and Loss of weight Index (FRAIL), and Edmonton Frail Scale (EFS), Modified Frailty Score (MFS), 12-item Short Form(SF-12), Tilburg Frailty Indicator (TFI).

**Table 1:** Characteristics of the included studies.

Reference	Country	Region	Study design	Sample size	Age (years)	hemodialysis duration	Diagnostic criteria	Prevalence of frailty (%)	Risk of bias
(15)	UK	Europe	Cohort	485	Median 63	Median 37 months	CFS	53.8	Low
(6)	Spain	Europe	Cohort	320	70.26 ± 13.85	Unclear	FP	5.6	High
Cai et al. (2019)	China	Asia	Cross-sectional	201	56.77 ± 15.24	Median 2 years	FRAIL	44.8	Low
Canton et al. (2019)	Spain	Europe	Cohort	277	Median 65	Median 34.6 months	EFS	29.6	Low
Chen et al. (2021)	China	Asia	Cross-sectional	156	Unclear	Unclear	FRAIL	71.8	Moderate
Chen et al. (2021)	China	Asia	Cross-sectional	467	Median 54	Median 35 months	FP	35.5	Low
(25)	China	Asia	Cohort	313	66.1 ± 12.5	Median 39 months	FP	40.3	Low
Chen et al. (2023)	China	Asia	Cross-sectional	303	53.74 ± 15.60	Unclear	FRAIL	12.2	Low
Chiang et al. (2018)	USA	America	Cohort	440	56.1 ± 14.2	Median 2.7 years	FP	28.9	Low
(16)	UK	Europe	Cohort	2089	64.6 ± 16.6	Median 29.8 months	CFS	42.6	Low
Du et al. (2018)	China	Asia	Cohort	380	63.76 ± 6.54	30.06 ± 7.18 months	FP	44.2	Low
Duan et al. (2019)	China	Asia	Cross-sectional	100	59.03 ± 16.14	Unclear	FRAIL	30.0	Moderate
Fan et al. (2021)	China	Asia	Cross-sectional	143	55.69 ± 13.74	Unclear	FRAIL	31.5	Moderate
Fitzpatrick et al. (2019)	USA	America	Cohort	370	54.9 ± 13.1	Median 3.4 months	FP	52.2	Low
(33)	USA	America	Cohort	285	55 ± 13	Median 3.4 months	FP	57.0	Low
(10)	China	Asia	Cohort	208	60.5 ± 12.7	Median 82 months	FP	25.4	Low
Gao et al. (2022)	China	Asia	Cross-sectional	141	51.01 ± 12.88	46.45 ± 34.66 months	FRAIL	9.2	Moderate
(34)	China	Asia	Cross-sectional	300	61.96 ± 13.68	Median 33 months	TFI	75.0	Low
(31)	China	Asia	Cohort	204	71.65 ± 5.89	Median 59 months	FP	72.1	Low
(12)	Japan	Asia	Cohort	2,404	61.6 ± 12.4	Median 3.8 years	SF-12	45.6	Low
(41)	UK	Europe	Cross-sectional	172	60.2 ± 16.5	Median 33.7 months	CFS	31.4	Moderate
Huang et al. (2020)	China	Asia	Cross-sectional	162	56.23 ± 13.67	Unclear	FRAIL	48.8	Moderate
Jafari et al. (2021)	Canada	America	Cross-sectional	109	63.3 ± 14.2	Median 34 months	FP	58.7	Low
Johansen et al. (2014)	USA	America	Cross-sectional	638	56.8 ± 14.57	Unclear	FP	29.8	Low
(29)	USA	America	Cohort	762	57.2 ± 14.2	Median 3.2 years	FP	31.5	Low
Johansen et al. (2018)	USA	America	Cohort	727	57.2 ± 14.3	Unclear	FP	31.6	Low
(44)	Korea	Asia	Cross-sectional	1,250	56.4 ± 13.2	5.1 ± 4.6 years	MFS	33.8	Low
Kimura et al. (2021)	USA	America	Cohort	337	56 ± 13	Unclear	FP	28.0	Low
(13)	USA	America	Cohort	745	57.1 ± 14.1 7	Unclear	FP	13.8	Low
Li et al. (2019)	China	Asia	Cross-sectional	439	60.65 ± 15.68	5.8 ± 4.25 years	FP	67.9	Moderate
Li et al. (2021)	China	Asia	Cohort	150	Median 69	Unclear	FP	34.7	High
Li et al. (2023)	China	Asia	Cross-sectional	389	56.94 ± 10.64	Unclear	FRAIL	33.7	Low
Liu et al. (2022)	China	Asia	Cross-sectional	150	Unclear	Unclear	TFI	73.3	Moderate
López-Montes et al. (2020)	Spain	Europe	Cohort	117	78.1 ± 4.1	Unclear	FP	53.8	Low
(50)	China	Asia	Cohort	761	65 ± 13	Median 39 months	FP	31.0	Low
McAdams et al. (2013)	USA	America	Cohort	146	60.6 ± 13.6	Unclear	FP	41.8	Moderate
McAdams et al. (2015)	USA	America	Cohort	324	54.8 ± 13.3	Unclear	FP	34.0	Low
(7)	Japan	Asia	Cohort	155	66.7 ± 14.1	Unclear	CFS	25.2	Moderate
(45)	Italy	Europe	Cohort	105	79.1 ± 7.6	Unclear	FI	55.2	Moderate
(40)	USA	America	Cohort	425	56.8 ± 13.3	Unclear	FP	29.4	Low
(38)	Japan	Asia	Cross-sectional	388	67.2 ± 11.9	Unclear	FP	21.4	Low
Turković et al. (2022)	Bosnia and Herzegovina	Europe	Cross-sectional	281	54.2 ± 11.91	60.5 ± 39.21 months	FP	44.8	Moderate
Wang et al. (2021)	China	Asia	Cross-sectional	176	52.2 ± 13.3	Unclear	FRAIL	6.3	High
Wang et al. (2021)	China	Asia	Cross-sectional	230	Unclear	Unclear	FRAIL	56.1	Low
(35)	China	Asia	Cross-sectional	185	55.6 ± 13.5	Unclear	TFI	38.9	Low
Wu et al. (2020)	China	Asia	Cross-sectional	168	Unclear	Unclear	FP	66.1	Moderate
Wu et al. (2020)	China	Asia	Cross-sectional	183	Unclear	Unclear	TFI	41.5	Moderate
Wu et al. (2021)	China	Asia	Cross-sectional	264	68.61 ± 7.59	Unclear	FP	36.0	Low
Xiao et al. (2023)	China	Asia	Cross-sectional	216	Unclear	Unclear	EFS	46.8	Moderate
Yadla et al. (2017)	India	Asia	cohort	205	44.95 ± 13.27	2.5 ± 1.3 years	FP	82.0	Low
Yang et al. (2020)	China	Asia	Cross-sectional	572	Unclear	Unclear	FRAIL	39.3	Low
Yang et al. (2022)	China	Asia	Cross-sectional	246	58.62 ± 10.09	Unclear	TFI	61.8	Moderate
Ye et al. (2018)	China	Asia	Cross-sectional	501	Unclear	Median 36 months	FP	43.1	Low
Ye et al. (2019)	China	Asia	Cross-sectional	237	Median 67	Median 35 months	FP	68.8	Moderate
(37)	China	Asia	Cross-sectional	503	53.02 ± 14.99	Unclear	FP	43.3	Low
Yoneki et al. (2018)	Japan	Asia	Cross-sectional	214	Mean 67.1	Unclear	FP	29.9	Moderate
(36)	China	Asia	Cross-sectional	187	53.2 ± 14.4	Unclear	FRAIL	5.9	Low
Zhang et al. (2022)	China	Asia	Cross-sectional	163	60.82 ± 13.51	Unclear	FRAIL	55.8	Moderate
Zhao et al. (2022)	China	Asia	Cross-sectional	129	Unclear	Unclear	FRAIL	30.2	Moderate
Zhao et al. (2022)	China	Asia	Cross-sectional	152	Unclear	Unclear	FRAIL	54.6	Moderate
Zhou et al. (2021)	China	Asia	Cross-sectional	315	Unclear	Unclear	TFI	41.9	Moderate
Zhu et al. (2017)	China	Asia	Cohort	186	Unclear	Unclear	FRAIL	37.6	Moderate
Zhu et al. (2022)	China	Asia	Cross-sectional	303	53.75 ± 15.59	Unclear	FRAIL	12.2	Low
Zhu et al. (2023)	China	Asia	Cross-sectional	146	67.45 ± 8.39	60.7 ± 44.72 months	FRAIL	18.5	Moderate

CFS: Clinical frailty scale; EFS: Edmonton Frail Scale; FP: Fried phenotype; FRAIL: Fatigue, Resistance, Ambulation, Illness and Loss of weight Index; FI: frailty index; MFS: Modified Frailty Score; SF-12: 12-item Short Form; TFI: Tilburg Frailty Indicator.

### 3.3 Methodological quality assessment

Regarding the overall risk of bias, the majority of the studies (*n* = 37, 57.8%) were classified as having a low risk of bias, 24 (37.5%) articles were identified as having a moderate risk of bias, and three (4.7%) as having a high risk of bias. Selection bias and non-response bias are the most common sources of bias in research. Selection bias was observed in 28 (43.8%) studies due to a lack of clarity about the sampling frame. Furthermore, the non-response rate was scarcely measured in the majority of studies. The risk of bias in the 64 included studies is shown in Table 1.

### 3.4 Prevalence of frailty among hemodialysis patients

**Figure 2:**
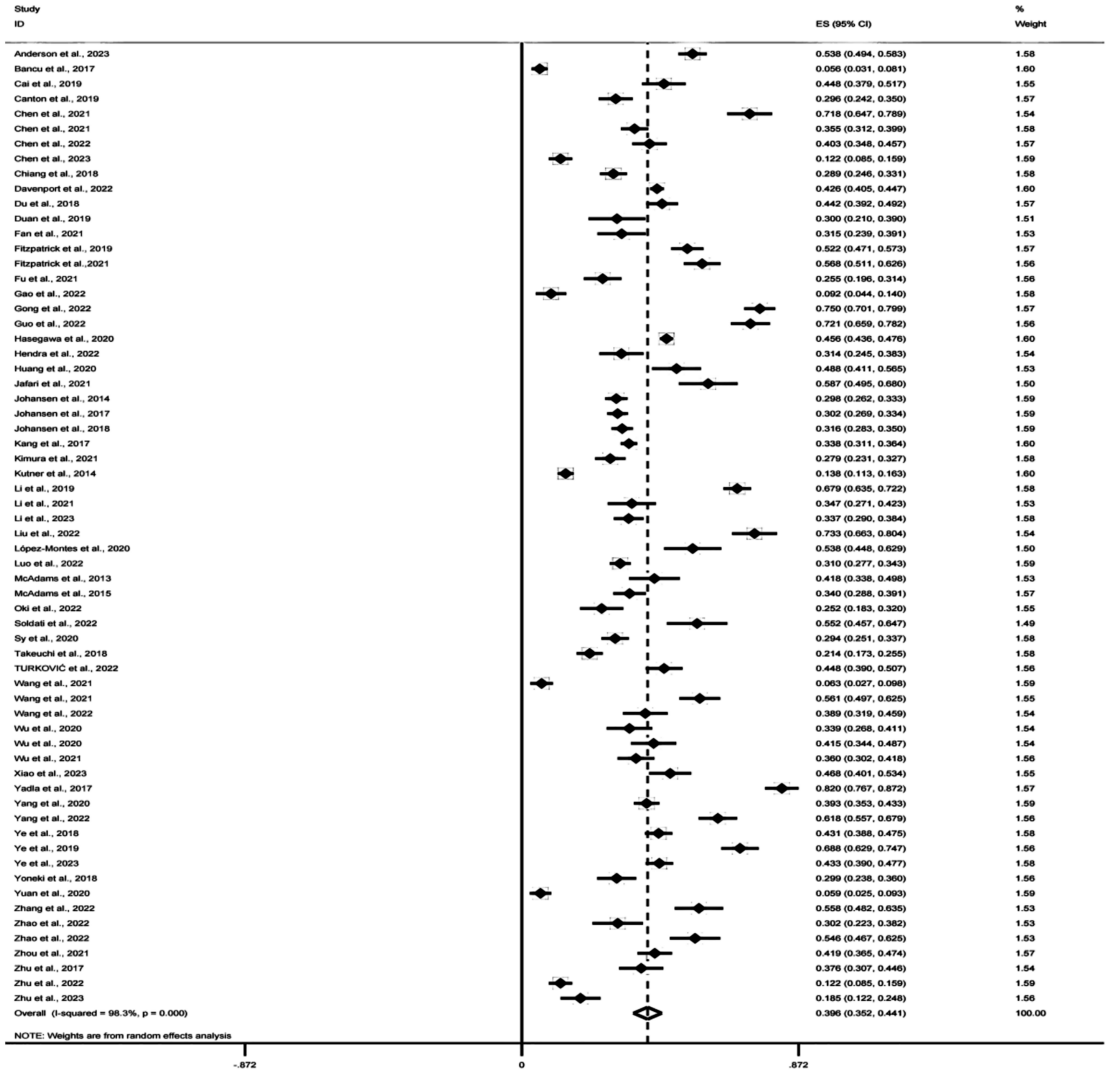
Forest plot overall frailty prevalence among hemodialysis patients.

The prevalence of frailty ranged from 5.6 to 82.0% in the 64 studies available for the meta-analysis. According to the random-effects model, it was estimated that the pooled prevalence of frailty among hemodialysis patients is 39.6% (95% *CI* 35–44%, *I*^2^ = 98.2%, *p* < 0.001). The meta-analysis results of frailty are presented in Figure 2.

### 3.5 Meta-regression analyses

The univariate and multivariate meta-regression analysis was performed to assess the association between the prevalence of frailty in hemodialysis patients and study characteristics, including sample size, age, duration of hemodialysis, gender, publication years, frailty criteria, and regions. The multivariate meta-regression model revealed that sample size (*β* = −0.038, 95% CI = −0.38 −0.03, *p* = 0.023), age (*β* = −0.438, 95% CI = 0.06–0.13, *p* = 0.018), and gender (*β* = 0.187, 95% CI = 0.03–0.17, *p* = 0.031) were significant potential moderators of the overall heterogeneity, yielding a model that was capable of explaining 33.8% of the variation between including studies (Table 2).

**Table 2:** Results of meta-regression analysis of frailty.

	Coefficient	*t*	*P*	95% CI	Adjusted *R*^2^
Univariate					
Sample size	−0.094	−2.15	0.036	(−0.18, −0.00)	5.52%
Age	0.335	3.15	0.034	(0.04, 0.63)	6.68%
Duration of hemodialysis	0.012	0.09	0.930	(−0.30, 0.32)	2.25%
Gender	0.095	2.00	0.049	(0.00, 0.19)	4.35%
Publication years	−0.009	−0.19	0.852	(−0.10, 0.84)	2.83%
Frailty criteria	0.022	1.48	0.143	(−0.00, 0.05)	3.87%
Regions	0.021	0.51	0.610	(−0.06, 0.10)	3.32%
Multivariate					
Sample size	−0.038	−1.31	0.023	(−0.38, −0.03)	33.8%
Age	0.438	3.35	0.018	(−0.06, 0.13)	
Duration of hemodialysis	0.084	0.32	0.631	(−0.27, 0.31)	
Gender	0.187	2.36	0.031	(0.03, 0.17)	
Publication years	−0.153	−0.56	0.384	(−0.12, 0.97)	
Frailty criteria	0.128	1.88	0.125	(−0.06, 0.10)	
Regions	0.034	0.68	0.523	(−0.19, 0.13)	

### 3.6 Stratified prevalence of frailty according to sample size, age, duration of hemodialysis, gender, publication years, frailty criteria, regions

Stratified analysis was employed to explore heterogeneity between studies (Table 3). In the subgroup analysis based on sample size, the estimates of pooled prevalence of frailty for sample sizes ≤300 and >300 were 43.7 and 34.3%, respectively. When assessed by age, the pooled prevalence for hemodialysis patients aged <60 and ≥60 was 29.2 and 53.2%, respectively. The pooled prevalence of frailty among patients on hemodialysis was 33.8% in males and 43.4% in females, and the values for patients with hemodialysis duration ≤1 and >1 year were 63.4 and 61.0%, respectively. In addition, we performed a subgroup analysis based on publication years, which identified that the prevalence of frailty in hemodialysis patients was 29.5% in pre-2017, 39.1% in 2017–2020, and 41.2% in 2021–2023. When analyzed using different evaluation scales, the incidence of frailty evaluation scales varied: FP, 40.8%; FRAIL, 33.1%; CFS, 38.6%; EFS, 38.1%; TFI, 55.5%; Other criteria, 38.5%. Finally, studies conducted in different geographical regions showed that the pooled prevalence of frailty in America, Asia, and Europe was 36, 41, and 39%, respectively.

**Table 3:** Subgroup analyses by the sample size, age, duration of hemodialysis, gender, publication years, frailty criteria, regions.

Subgroups	Number of included studies	Frailty prevalence	95% *CI*	*I^2^*	*P* value
Sample size					
≤ 300	37	43.7%	35.0–52.5%	98.2%	<0.001
> 300	27	34.3%	28.5–40.0%	98.5%	<0.001
Age					
<60 years	5	29.2%	8.6–49.8%	98.7%	<0.001
≥60 years	19	53.2%	44.0–62.3%	97.4%	<0.001
Duration of hemodialysis					
≤1 year	3	63.4%	38.6–88.1%	95.5%	<0.001
>1 year	7	61.0%	46.4–75.5%	96.2%	< 0.001
Gender					
Male	39	33.8%	28.2–39.5%	97.1%	<0.001
Female	38	43.4%	35.0–51.7%	98.0%	<0.001
Publication years					
<2017	4	29.5%	17.3–41.7%	97.1%	<0.001
2017 ~ 2020	25	39.1%	31.9–46.3%	98.6%	<0.001
2021 ~ 2023	35	41.2%	35.0–47.3%	98.0%	<0.001
Frailty criteria					
FP	31	40.8%	34.2–47.4%	98.4%	<0.001
FRAIL	18	33.1%	24.1–42.1%	98.1%	<0.001
CFS	4	38.6%	28.5–48.8%	95.0%	<0.001
EFS	2	38.1%	21.3–54.9%	93.5%	<0.001
TFI	6	55.5%	41.8–69.1%	96.6%	<0.001
Other criteria	3	38.5%	27.1–49.9%	97.9%	<0.001
Regions					
America	12	35.9%	28.8–43.0%	97.0%	<0.001
Asia	44	40.7%	34.9–46.4%	98.4%	<0.001
Europe	8	39.5%	24.7–54.2%	99.0%	<0.001

### 3.7 Publication bias and sensitivity analysis

Funnel plot asymmetry revealed evidence of publication bias in the included studies (Supplementary Figure S1). Similarly, the results of Egger’s test (*t* = 2.85, *p* = 0.006) also further confirmed the publication bias for the prevalence of frailty in hemodialysis patients. Therefore, we conducted a trim and fill analysis to adjust for this bias. During the analysis, studies included in frailty estimation among hemodialysis patients were adjusted, but no studies were found to be missing. The corrected pooled prevalence estimate of frailty was 39.6% (95% CI 3.52–4.41%), which is similar to the unadjusted prevalence of frailty, indicating the results of the meta-analysis are valid (Supplementary Figure S2). Additionally, the results of the sensitivity analysis showed that the pooled prevalence of frailty was not significantly altered even after the removal of any single study, further illustrating the robustness of meta-analysis findings (Supplementary Figure S3).

## 4 Discussion

### 4.1 Summary of findings

This systematic review and meta-analysis were conducted to estimate the prevalence of frailty in hemodialysis patients from a global perspective. The results of the meta-analysis revealed the global prevalence of frailty in hemodialysis patients was 39.6%, which is lower than the systematic review published in 2021 (19). The source of this discrepancy could be explained by our rigorous inclusion and exclusion criteria, wider subject population and larger sample size. However, there was considerable heterogeneity among the included studies, and meta-regression did not reveal any significant differences, except for sample size, age and sex. The inability of most covariates to explain the heterogeneity is a significant finding. It highlights that frailty in hemodialysis is a multifaceted syndrome whose prevalence is shaped by a wide array of clinical, methodological, and contextual factors, rather than by simple demographics. This inherent variability argues against a universal prevalence estimate and strongly supports the standardized assessment of frailty in clinical practice to guide individualized care. It’s worth noting that the overall prevalence of frailty (39.6%) in patients on hemodialysis was higher compared to non-dialysis patients (21.1%) (24). The high prevalence of frailty in hemodialysis patients may be explained by the fact that dialysis accelerates the loss of nutrients such as proteins and amino acids, which in turn leads to catabolism of muscle tissue and energy depletion, causing a decline in muscle mass and physical function (25–27). In addition, post-dialysis fatigue is a common frailty symptom in hemodialysis patients and is associated with an increase in inflammatory cytokines and a reduction in creatinine and serum albumin, which have a negative impact on sleep and rest, leading to frailty (28, 29). Therefore, early diagnosis of frailty in hemodialysis patients is a crucial factor in preventing the risk of adverse events including disability and mortality, as well as improving quality of life.

Although studies with sample sizes of less than 100 were excluded, based on our subgroup analysis, we still found that the prevalence of frailty in smaller sample groups (43.7%) was significantly higher than in larger sample groups (34.3%). In general, a smaller sample size may increase the risk of selection and publication bias, leading to more extreme prevalence estimates (30). Thus, it is essential for researchers to give careful consideration to how best to sample to minimize selection bias in their future studies.

The stratified analysis by age demonstrated that the prevalence of frailty was higher in older hemodialysis patients (53.2%) than in younger ones (29.2%), consistent with recent studies (19, 31). When frailty is considered purely in physical terms, age is most strongly correlated with frailty (30). It is widely acknowledged that physical functions and metabolism decline with age, which greatly increases the risk of frailty, especially for hemodialysis patients. Prior investigations have also substantiated the strong correlation between aging and frailty in hemodialysis patients (32, 33). In particular, malnutrition and fatigue brought on by sustained hemodialysis treatment may also accelerate the aging process in hemodialysis patients (34–36). Consequently, healthcare professionals should pay attention to the frailty of elderly hemodialysis patients and provide early intervention to slow down the frailty process.

Through subgroup analysis of hemodialysis duration, we found that the pooled prevalence of frailty is higher in patients with hemodialysis duration ≤1 year than in those with >1 year. This was consistent with a study conducted in China on frailty in hemodialysis patients (37). The initiation of dialysis stands out as one of the most arduous stages for patients undergoing hemodialysis treatment. First of all, many ESRD patients have multiple comorbidities before hemodialysis, such as diabetes, hypertension, and cognitive dysfunction, which cannot be effectively treated with hemodialysis alone and may contribute to frailty [(38); McAdams et al., 2015]. Secondly, the pathophysiological and psychological burdens associated with the initial phase of hemodialysis may exacerbate the symptoms of uremia, such as fatigue, edema, malnutrition, and anemia, which may adversely affect patients (39, 40).

Analysis of gender subgroups indicated that women were more likely than men to experience frailty among hemodialysis patients, which was in line with the results of a previous review (19). This result could potentially be attributed to the fact that females generally exhibit lower physiological muscle mass and body mass index (BMI), and the correlation between BMI, sarcopenia, and frailty has been substantiated in prior researches (33, 41). Additionally, an alternative explanation is that postmenopausal women have lower testosterone as a result of multiple anabolic hormone deficiencies, and declining testosterone levels are strongly associated with frailty (42, 43). There is a clear need for healthcare professionals to further explore gender differences in the frailty status of hemodialysis patients, which may be beneficial in developing individualized treatment plans for patients to achieve better health outcomes.

The result of the publication years-stratified revealed that the pooled prevalence of frailty in hemodialysis patients is higher in 2021 to 2023 (41.2%) compared to that in pre-2017 (29.5%) and 2017 to 2020 (39.1%). The potential cause for this trend is the growing attention toward frailty as the population ages. Furthermore, advances in precision medicine technology and an increase in relevant studies are gradually confirming the high prevalence and adverse consequences of frailty in hemodialysis patients (44, 45). Nevertheless, the intricate interaction mechanism between hemodialysis and frailty remains unclear and warrants in-depth exploration.

At present, there is a lack of universally accepted standard tool or diagnostic criteria for assessing frailty. Subgroup analyses revealed that the pooled prevalence of frailty varied according to the assessment tool used. In all eligible studies, the most frequently employed tools for measuring frailty were FP and FRAIL scales. The main advantages of FP and FRAIL scales are the relative simplicity of application and the minimal amount of data required for computation, making them a potential tool for screening (46, 47). The highest pooled prevalence of frailty in hemodialysis patients was reported when the diagnostic criteria were restricted to the Tilburg Frailty Indicator (55.5%). The TFI is a self-reporting scale that may be influenced by patients’ subjective perceptions, leading to overestimating the prevalence of frailty. In addition, a previous study has revealed the poor sensitivity and specificity of the TFI for screening frailty among hemodialysis patients (48). Hence, clinical researchers should strike a balance between simplicity and reliability when choosing a tool to assess frailty.

To date, there is no consensus on the global prevalence of frailty in hemodialysis patients. Subgroup analysis revealed that the prevalence of frailty varied by geographic regions, with higher rates in Asia (40.7%) compared with that in Europe (39.5%) and America (35.9%). This discrepancy may be due to that the included studies were mainly conducted in Asian countries, which is the region with the highest number of ESRD patients worldwide (49), resulting in a higher overall prevalence of frailty in Asia. Furthermore, the higher prevalence of frailty in Asian hemodialysis patients may also be attributed to poorer economic status, health literacy, and access to healthcare (50, 51). Thus, healthcare professionals should further explore the differences in the prevalence of frailty among hemodialysis patients between various geographical regions in their future studies.

### 4.2 Strengths and limitations

Overall, this systematic review has several strengths. To the best of our knowledge, this is the first systematic review to comprehensively analyze the pooled prevalence of frailty in hemodialysis patients from a global perspective. We carried out an extensive search strategy across multiple electronic databases and applied a rigorous approach to study selection, data extraction, and appraisal. In addition, this meta-analysis included a larger number of studies than previous meta-analyses, provided the pooled prevalence of frailty in seven different subgroups of hemodialysis patients, and examined the potential moderators to help explain the detected statistical heterogeneity. Finally, sensitivity analyses were undertaken to evaluate the reliability of the main findings.

Despite the many strengths of the current study, several limitations should be taken into consideration. Firstly, the restriction of the search to English and Chinese literature may introduce a potential limitation, as it may have introduced language bias and compromised the global scope of this review through the possible omission of relevant studies from other linguistic regions (such as European and Latin American countries). Secondly, notable heterogeneity was observed in the meta-analysis of prevalence. Except for sample size, age, and gender, other factors did not yield conclusive evidence to determine the possible causes of heterogeneity. Fourthly, our study excluded valuable data from smaller or resource-limited settings (those with sample sizes below 100), which may affect the global representativeness of our findings. Finally, although sensitivity analyses showed that the results of the meta-analysis were reliable, there may be a publication bias because unpublished studies were not considered.

## 5 Conclusion

In summary, this systematic review demonstrated that the global prevalence of frailty in hemodialysis patients was 39.6% and varied significantly by the sample size, age, duration of hemodialysis, gender, publication years, frailty criteria, and regions. These results suggest that frailty management of hemodialysis patients should focus on the risk factors affecting this condition, especially on controllable factors such as malnutrition and fatigue, and effective prevention and intervention strategies should be considered in future research.

## Data Availability

The original contributions presented in the study are included in the article/Supplementary material, further inquiries can be directed to the corresponding author/s.
