# Supplementary material for: Global prevalence of frailty in hemodialysis patients: a systematic review and meta-analysis
Source: Front Med (Lausanne). 2025 Dec 16;12:1722657. doi: 10.3389/fmed.2025.1722657 (PMC12748182; doi:10.3389/fmed.2025.1722657)
Supplement: Supplementary file 1 [file Table_1.DOCX]

**supplementary section**


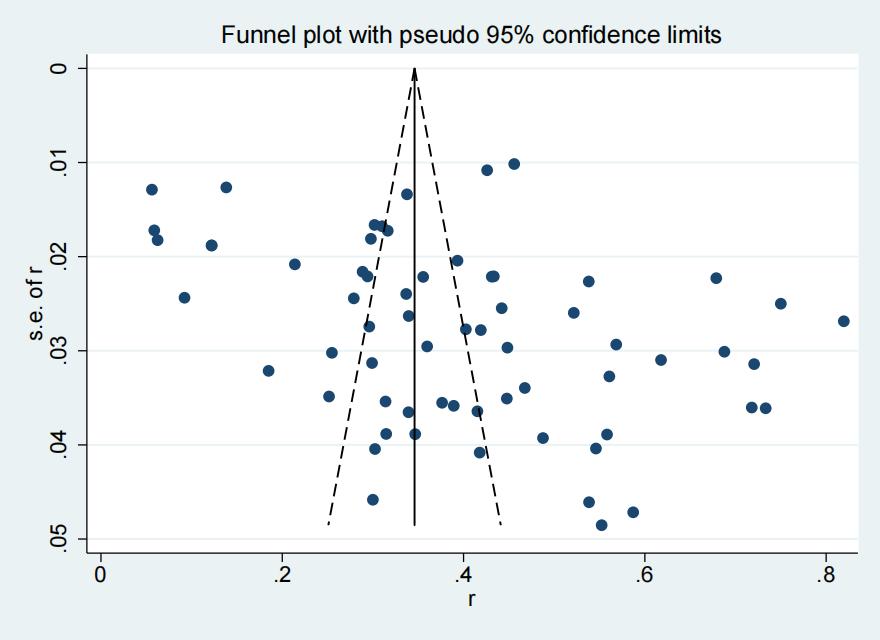


**Figure S1. Funnel plot for assessing publication biases**


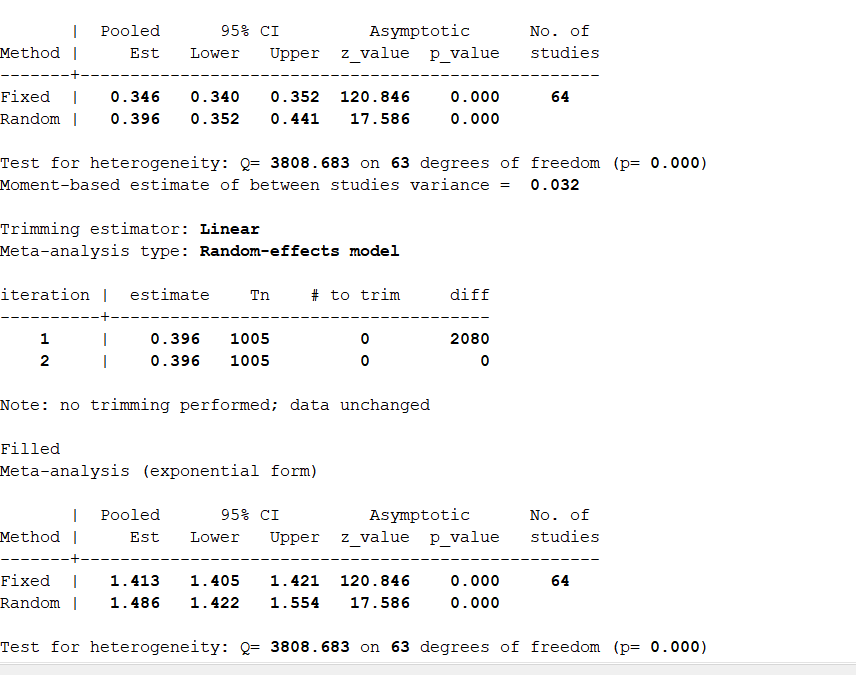


**Figure S2. Trim-and-fill analysis for the prevalence of frailty**


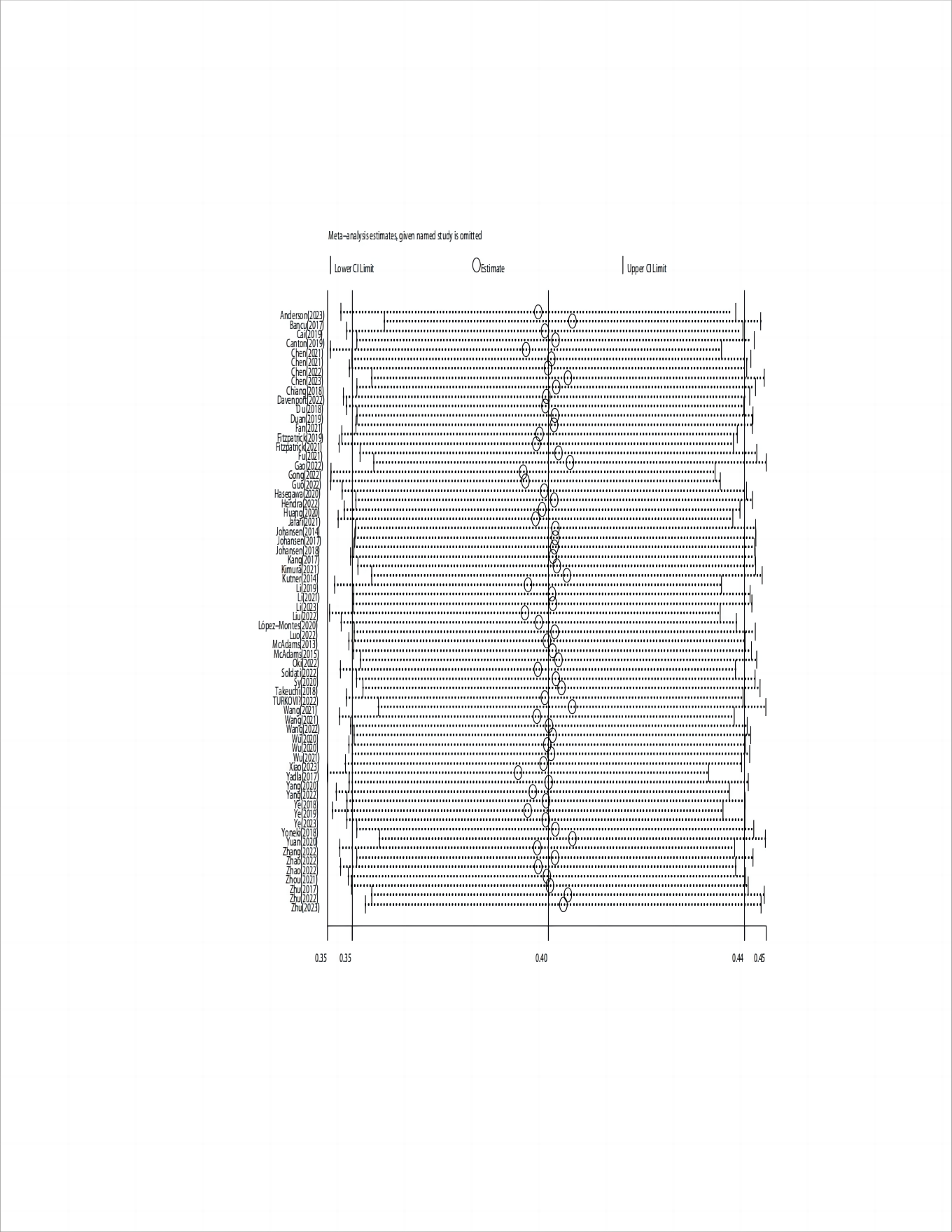


**Figure S3. Sensitivity analysis for the prevalence of frailty**

**、**
