# Supplementary material for: Global prevalence of frailty in hemodialysis patients: a systematic review and meta-analysis
Source: Front Med (Lausanne). 2025 Dec 16;12:1722657. doi: 10.3389/fmed.2025.1722657 (PMC12748182; doi:10.3389/fmed.2025.1722657)
Supplement: Supplementary file 2 [file Table_2.DOCX]

**supplementary section (S1)**

1. **Cochrane Library Search Strategy**

Search Date: June 15, 2023

Final Search:#8

| **Search** | **Query** | **No. of Results** |
| --- | --- | --- |
| **#1** | MeSH descriptor: [dialysis] explode all trees | 19563 |
| **#2** | (“dialysis”):ti,ab,kw OR (“hemodialysis”):ti,ab,kw | 21773 |
| **#3** | #1 OR #2 | 24090 |
| **#4** | MeSH descriptor: [Frailty] explode all trees | 3268 |
| **#5** | MeSH descriptor: [Frail Elderly] explode all trees | 2337 |
| **#6** | (“frail”):ti,ab,kw OR (“frailty”):ti,ab,kw OR (“frailties”):ti,ab,kw OR (“fragility”):ti,ab,kw OR (“frailty syndrome”):ti,ab,kw | 6756 |
| **#7** | #4 OR #5 OR #6 | 7013 |
| **#8** | #3 AND #7 | 131 |

1. **Medline Search Strategy**

Search Date: June 15, 2023

Final Search: S3

| **Search** | **Query** | **No. of Results** |
| --- | --- | --- |
| **S1** | SU dialysis OR SU hemodialysis | 145768 |
| **S2** | SU frailty OR SU frail OR SU frailties OR SU fragility OR SU frailty syndrome | 31174 |
| **S3** | S1 AND S2 | 398 |

1. **CINAHL Search Strategy (CINAHL Plus with Full Text)**

Search Date: June 15, 2023

Final Search: S3

| **Search** | **Query** | **No. of Results** |
| --- | --- | --- |
| **S1** | SU dialysis OR SU hemodialysis | 31033 |
| **S2** | SU frail OR SU frailty OR SU frailties OR SU fragility ORSU frailty syndrome | 12521 |
| **S3** | S1 AND S2 | 103 |

1. **PubMed Search Strategy**

Search Date: June 15, 2023

Final Search: #7

| **Search** | **Query** | **No. of Results** |
| --- | --- | --- |
| **#1** | "dialysis"[Mesh Terms] | 24106 |
| **#2** | "dialysis"[Title/Abstract] OR "hemodialysis"[Title/Abstract] | 165848 |
| **#3** | #1 OR #2 | 183882 |
| **#4** | "frailty"[MeSH Terms] | 8903 |
| **#5** | "frail"[Title/Abstract] OR "frailty"[Title/Abstract] OR "frailties"[Title/Abstract] OR "fragility"[Title/Abstract] OR "frailty syndrome"[Title/Abstract] | 54954 |
| **#6** | #4 OR #5 | 55336 |
| **#7** | #3 AND #6 | 903 |

1. **Embase Search Strategy**

Search Date: June 15, 2023

Final Search: #11

| **Search** | **Query** | **No. of Results** |
| --- | --- | --- |
| **#1** | 'dialysis'/exp | 335973 |
| **#2** | 'hemodialysis'/exp | 137894 |
| **#3** | 'dialysis':ti,ab,kw OR 'hemodialysis':ti,ab,kw | 247773 |
| **#4** | #1 OR #2 OR #3 | 353714 |
| **#5** | 'frailty'/exp | 25578 |
| **#6** | 'fragility'/exp | 25 |
| **#7** | 'frailty syndrome'/exp | 83 |
| **#8** | 'frail elderly'/exp | 12135 |
| **#9** | 'frail':ti,ab,kw OR 'frailty':ti,ab,kw OR 'frailties':ti,ab,kw OR 'fragility':ti,ab,kw OR 'frailty syndrome':ti,ab,kw | 82743 |
| **#10** | #5 OR #6 OR #7 OR #8 OR #9 | 89065 |
| **#11** | #4 AND #10 | 2038 |

1. **Web of Science Search Strategy**

Search Date: June 15, 2023

Final Search: #3

| **Search** | **Query** | **No. of Results** |
| --- | --- | --- |
| **#1** | TS=("dialysis" OR "hemodialysis") | 389738 |
| **#2** | TS=("frailty" OR "frail" OR "frailties" OR "fragility" OR "frailty syndrome") | 111236 |
| **#3** | #1 AND #2 | 1782 |

1. **Scopus Search Strategy**

Search Date: June 15, 2023

Final Search: #3

| **Search** | **Query** | **No. of Results** |
| --- | --- | --- |
| **#1** | TITLE-ABS-KEY ( "dialysis"  OR  "hemodialysis" ) | 286146 |
| **#2** | TITLE-ABS-KEY ( "frail"  OR  "frailty"  OR  "frailties"  OR  "fragility"  OR  "frailty syndrome") | 114831 |
| **#3** | #1 AND #2 | 1505 |

**H. CNKI Search Strategy**

Search Date: June 15, 2023

| [(主题="血液透析" + "维持性血液透析" + "常规血液透析" + "间断性血液透析" + "血液净化治疗") AND (主题="衰弱" + "虚弱" + "衰弱症" + "衰弱综合征")](https://kns.cnki.net/KNS8/AdvSearch?id=615&dbcode=SCDB&searchtype=gradeSearch&ishistory=1" \t "_blank" \o "(主题="乳腺癌" + "乳房癌" + "乳房肿瘤" + "乳腺肿瘤"  + "乳腺恶性肿瘤") AND (主题="衰弱" + "虚弱" + "衰弱症" + "衰弱综合征"))/85 |
| --- |

**I. VIP Search Strategy**

Search Date: June 15, 2023

| [(((((题名或关键词="血液透析" OR 题名或关键词="维持性血液透析") OR 题名或关键词="常规血液透析") OR 题名或关键词="间断性血液透析") OR题名或关键词="血液净化治疗") AND (((题名或关键词="衰弱" OR 题名或关键词="虚弱") OR 题名或关键词="衰弱症") OR 题名或关键词="衰弱综合征"))](http://lib.cqvip.com/Qikan/search/index?LngMySearHistoryIdGuid=35f85ed6-00e0-4eee-b999-17896f9298ec&from=Qikan_Article_History)/**77** |
| --- |

**J. WAN FANG Search Strategy**

Search Date: June 15, 2023

| 题名或关键词:(血液透析 OR 维持性血液透析 OR 常规血液透析 OR 间断性血液透析 OR 血液透析治疗) and 题名或关键词:(衰弱 OR 虚弱 OR 衰弱症 OR 衰弱综合征))/85 |
| --- |

**K. CBM Search Strategy**

Search Date: June 15, 2023

| [("血液透析"[常用字段:智能] OR "维持性血液透析"[常用字段:智能] OR "常规血液透析"[常用字段:智能] OR "间断性血液透析"[常用字段:智能] OR "血液透析治疗"[常用字段:智能]) AND( "衰弱"[常用字段:智能] OR "虚弱"[常用字段:智能] OR "衰弱症"[常用字段:智能] OR "衰弱综合征"[常用字段:智能])](javascript:toDoRelimitSearch();)/129 |
| --- |
